# Insights into the gel and electronic sense characteristics of meat batters made from Funiu white goat and Oula sheep meat in different rigor states

**DOI:** 10.1016/j.fochx.2022.100523

**Published:** 2022-11-26

**Authors:** Shaobo Li, Dequan Zhang, Can Xiang, Yue Ge, Huan Liu, Xiaochun Zheng, Li Chen, Zhenyu Wang

**Affiliations:** Institute of Food Science and Technology, Chinese Academy of Agricultural Sciences/Key Laboratory of Agro-Products Processing, Ministry of Agriculture, Beijing 100193, China

**Keywords:** Meat batters, Pre-rigor meat, Post-rigor meat, Gel properties, Electronic sense

## Abstract

To study the effect of rigor state on physicochemical characteristics of Funiu white goat and Oula sheep meat batters. In this study, the effect of rigor state of Funiu white goat and Oula sheep silverside muscle on meat batter quality and physicochemical properties was determined. Results indicated that meat batters obtained from post-rigor meat had less water holding capacity and lower immobilized water than that obtained from pre-rigor meat. The gel network of meat batters produced with Funiu white goat muscle appeared denser and smoother compared with Oula sheep meat batters. Furthermore, meat batters produced with pre-rigor meat had higher hardness compared to that produced with post-rigor meat. Differences in response values were found for CPS (sensitivity to sweet taste) of meat batters made with meat in the three rigor states. Collectively, both Funiu white goat and Oula sheep meat in pre-rigor state were more suitable to minced meat product production compared to post-rigor meat based on gel properties.

## Highlights

• Meat batters produced with pre-rigor meat had better gel properties than that of meat batters produced with rigor meat.
• Funiu white goat and Oula sheep meat batters had distinct odor quality profiles.
• Meat batters produced with pre-rigor, rigor and post-rigor meat had distinct taste sense.

## Introduction

Goat and sheep meat are good sources of nutrition (Banerjee et al., 2012, Holman et al., 2015). In recent years, a variety of food products based on goat and sheep meat have reached the market, including smoked, roasted, cured, and minced meat products (Cunha et al., 2018, Teixeira et al., 2020). Particularly, minced meat products, including meatballs and sausages, have become increasingly popular among consumers due to their taste and convenience (Oz, 2021).

Minced meat products represent a compact matrix rich in protein, water, and lipid, which show excellent gel and viscoelastic properties. The quality of minced meat products is decided by the intrinsic properties of raw material, grinding, and thermal treatment (Wrona, Nerin, Alfonso, & Caballero, 2017). It is challenging to assess the quality of minced meat products because of the complexity of the matrix. Therefore, meat batter is often used as a model to explore the influence of different factors on the quality of minced meat products (Zhao et al., 2019). As the largest producer of goat meat and sheep meat worldwide, China has a great selection of corresponding finished products, including meatballs and dumplings. However, information regarding the physicochemical characterization of goat and sheep meat minced products is limited.

Rigor mortis and ageing of meat are two biochemical stages that animal must go through after slaughter (Reid et al., 2017). Many studies have demonstrated that palatability of meat in the rigor mortis state is worse than that of aged meat. Xiao et al. (2020) verified that roasted lamb produced with post-rigor meat had lower shear force than roasted lamb produced with meat during rigor mortis. Moreover, hot-fresh meat is widely popular in China due to its health benefits and texture properties. Hot-fresh meat has not yet undergone rigor mortis and can be considered as pre-rigor meat. Aged meat can be considered as post-rigor meat because it has experienced the complete process of rigor mortis (Li et al., 2022). Understanding the quality characteristics of pre- and post-rigor meat is important for developing improved meat products. However, the texture, moisture mobility, and sensory characteristic of goat and sheep meat batters produced with meat in pre- and post-rigor states are not clearly understood.

Hence, the work aimed to study the effect of rigor state on physicochemical characteristics of Funiu white goat and Oula sheep meat batters. The study provided some new information about the differences in terms of quality of meat batters produced with the meat in different rigor states, thus contributing to improve the quality of minced meat products produced with goat and sheep meat.

## Materials and methods

### Sample preparation

Ten male Funiu white goats and ten male Oula sheep were included in this study. Funiu white goats were obtained from Xianchang Holdings Co. Ltd. (Henan, China). Animals were slaughtered using standard routines of the commercial slaughter-house. Samples were obtained from both left and right sides of silverside muscles immediately and chilled at 4 °C, and divided into three sample groups based on time elapsed after slaughtering. The pre-rigor goat meat (G1), rigor goat meat (G2), and post-rigor goat meat (G3) were collected at 45 min, 24 h, and 72 h after slaughter, respectively (Li et al., 2022). All samples were quickly frozen in a freezer warehouse. Oula sheep were obtained from Xiangsanjiang Animal Husbandy Development Co. Ltd. (Qinghai, China). The pre-rigor sheep meat (S1), rigor sheep meat (S2), and post-rigor sheep meat (S3) from Oula sheep were collected as same as the samples from Funiu white goat. Collected samples were kept at −20 °C until analysis.

### Meat batters preparation

It removed the adipose and connective tissues of meat samples after thawing. Meat samples originated from each muscle and each animal were employed separately to prepare meat paste. Then, meat samples of each group were cut into 2-cm cubes which were ground for 1.5 min in a grinder. The sodium chloride and cold water were added to the meat paste at a proportion of 2.5 % and 10 %, respectively. Then, meat paste was ground for additionally 1.5 min. Aliquots of 40 g of meat batters of each sample group were placed into cylindrical centrifugal tubes, and sample weight was measured (*w_1_*), after which samples were then treated differently, i.e., samples destinated to rheological testing were stored at 4 °C for 5 h. The remaining samples were centrifuged at 1000*g* for 1.5 min at 4 °C to remove air, heated at 80 °C for 20 min in a water bath, and formed gels were cooled for 20 min in ice water followed by storage at 4 °C overnight in a refrigerator for other physicochemical property measurements. Three independent meat batter samples for each treatment, and 3 replicates for each sample were used.

### Chemical composition analysis

The contents of moisture, protein, lipid, and ash of meat batter samples were measured using the methods of AOAC (AOAC, 2000).

### pH analysis

pH analysis was determined using the method of Li, He, and Li (2018). Briefly, four grams cooked meat batter were blended with 40 mL potassium chloride（0.1 mol/L, pH 7.0) using a homogenizer (8000 rpm, 30 s). Then, the mixture was used to pH values measurement by a digital pH meter.

### Color analysis

The lightness (L*), redness (a*), and yellowness (b*) of samples were determined by a colorimeter. Each sample was measured at four random locations. Whiteness (*W*) was calculated by the method of Li, He, and Li (2018) as follows: *W* = L* − 3b*.

### Water holding capacity analysis

Cooking loss of samples were measured as described by Li, He, and Li (2018). Briefly, meat batters from tubes were removed after standing for 35 min at room temperature. Then, it measured the weights of meat batters (*w_2_*) after removing the water on the surface using filter paper. Cooking loss was calculated by the following equation:

$$\mathrm{Cooking}\;loss\left(\%\right)=\left(w_{1}-w_{2}\right)/w_{1}\times 100$$

Centrifugal loss of each sample was determined by the method of Li, He, and Li (2018). About 50 g (*m*_1_) of meat batter sample were wrapped by multi-layer filter paper and put into a 100-mL centrifuge tube. Then, meat batters were centrifuged at 3200*g* for 15 min. The weight of meat batters after removing filter paper was recorded as *m*_2_. The centrifugal loss was measured by the following formula:

$$\mathrm{Centrifugal}\;loss\left(\%\right)=\left(m_{1}-m_{2}\right)/m_{1}\times 100$$

### LF NMR analysis

The LF NMR relaxations of cooked meat batters were determined using the method of Li et al. (2022). The cooked meat batters cylinder of each sample was cut into about 20-mm height and was placed in NMR tubes. Twenty scans were got at 3 s intervals for each sample.

### Texture analysis

The texture properties of cooked meat batters were measured using the method of Wu, He, Hong, and Wang (2016) using a texture analyzer (Brookfield, USA). The cooked meat batters cylinders of each sample were cut into about 25 mm height. The equipment parameters were set as follows: axially compressed 50 %, test speed 1.0 mm/s, post-test speed 1.0 mm/s, trigger type 5 g.

### Microstructure analysis

Sample for observing microstructure was prepared by the method of Li et al. (2020). The cooked meat batters were cut into about 2 × 2 × 2 cm^3^ cubes and fixed with 10 mL of glutaraldehyde (2.5 %, pH 6.8) for 24 h. The cubes were washed three times by phosphate buffer (pH 6.8, 0.1 mol/L) for 10 min. Then, the cubes were dehydrated in different concentration ethanol solution three times successively. The cubes were vacuum freeze dried after replaced with *tert*-butanol. The microstructure of cooked meat batters were measured by scanning electron microscope (SEM) under 2000× magnification.

### Rheological analysis

Rheological characteristics of meat batters were measured by the method of Westphalen, Briggs, and Lonergan (2006). The equipment (DHR-1, TA, USA) parameters were slit-width 1.0 mm, oscillation frequency 1 Hz, and clamp distance 40 mm.

### Electronic nose (E-nose) analysis

E-nose analysis using an Odour Sensing System E-nose (PEN 3.5, Airsense, Germany). The system was installed with 10 chemical sensors: W1W (sulphur-organic), and W2W (sulph-chlor), W1C (aromatic), W3C (aromatic), W5C (arom-aliph), W1S (broad-methane), W2S (broad-alcohol), W5S (broad range), W3S (methane-aliph), W6S (hydrogen).

### Electronic tongue (E-tongue) analysis

Five grams of each cooked meat batters were ground and then put into a 250 mL beaker with 150 mL water. After homogenizing, the mixture was extracted by ultrasonic for 20 min. After that, the mixture was centrifuged at 8000*g* for 20 min at 4 °C. The supernatant was filtered by 0.45 μm filter membrane before analysis using a Taste Sensing System E-tongue (Asrree II, Alpha, France). The system was installed with 3 chemical sensors for fresh taste (NMS), sourness (AHS), and saltiness (CTS), and 4 universal sensor SCS (sensitive to bitterness) and CPS, ANS, and PKS (sensitive to sweet taste).

### Statistical analysis

The data were analyzed based on a general linear model of analysis of variance (ANOVA) by SPSS 22.0 (IBM Corp., NY, USA). The rigor state and animal type were considered as fixed effects, meanwhile the animal and silverside muscles were considered as random effects. The significant difference between means was contrasted by Duncan’s multiple range test (*P* < 0.05).

## Results and discussion

Main effect and interaction effect of two factors on the physicochemical properties of meat batters are presented in Table S1.

### Chemical composition

Table 1 shows nutritional characteristics of meat batters made from Funiu white goat and Oula sheep meat in different rigor states. Moisture content was lower in meat batters made from rigor meat than that made from pre-rigor meat. Because the organizational structure of pre-rigor meat was complete, and it was important for water holding capacity of meat batters (Balan et al., 2019). Funiu white goat meat batters had higher moisture content than Oula sheep meat batters, and fat content in Funiu white goat meat batters was lower than that in Oula sheep meat batters. Furthermore, fat content was higher in meat batters made from rigor meat than that made from pre-rigor meat. Ash content determined in meat batters made from Funiu white goat and Oula sheep meat in different rigor state showed no significant difference.

**Table 1:** Nutritional characteristics (g/100 g meat) of meat batters made from Funiu white goat and Oula sheep meat in different rigor states (n = 9).

	Funiu white goat			Oula sheep		
	Pre-rigor	Rigor	Post-rigor	Pre-rigor	Rigor	Post-rigor
Moisture	74.40 ± 0.35^Aa^	73.56 ± 0.36^Ab^	74.08 ± 1.47^Aab^	73.66 ± 0.30^Ba^	72.53 ± 0.66^Aa^	72.66 ± 0.54^Aa^
Protein	22.50 ± 0.88^Aab^	23.96 ± 0.63^Aa^	21.27 ± 0.93^Ab^	22.31 ± 0.90^Aa^	24.28 ± 1.12^Aa^	22.73 ± 0.72^Aa^
Fat	1.55 ± 0.18^Bb^	2.13 ±0.36^Ba^	2.03 ± 0.37^Aa^	2.22 ± 0.18^Ab^	2.66 ± 0.36^Aa^	2.36 ± 0.39^Aab^
Ash	1.25 ± 0.21^Aa^	1.59 ± 0.36^Aa^	1.42 ± 0.35^Aa^	1.54 ± 0.20^Aa^	1.75 ± 0.10^Aa^	1.55 ± 0.20^Aa^

Values with different lowercase letters in the same animal type are significantly different;

Values with different uppercase letters in the same rigor state are significantly different.

The results are presented as means and standard errors.

Protein content can significantly affect meat batters’ gel formation, which is crucial for the quality of meat batters. Protein content can influence consumers’ acceptance of products because of its influence on color, tenderness, and juiciness of the meat batters products (Spencer, Cienfuegos, & Guinard, 2018). In this study, the protein content of Funiu white goat meat batters was similar to Oula sheep meat batters. Furthermore, protein content was significantly higher in meat batters made from rigor meat than that made from pre-rigor meat. It may be because the meat batters produced with pre-rigor meat had better gel properties than that produced with rigor meat, and it caused the higher moisture content in pre-rigor meat batters (Choi et al., 2015, Ge et al., 2021).

### pH

**Fig. 1:**
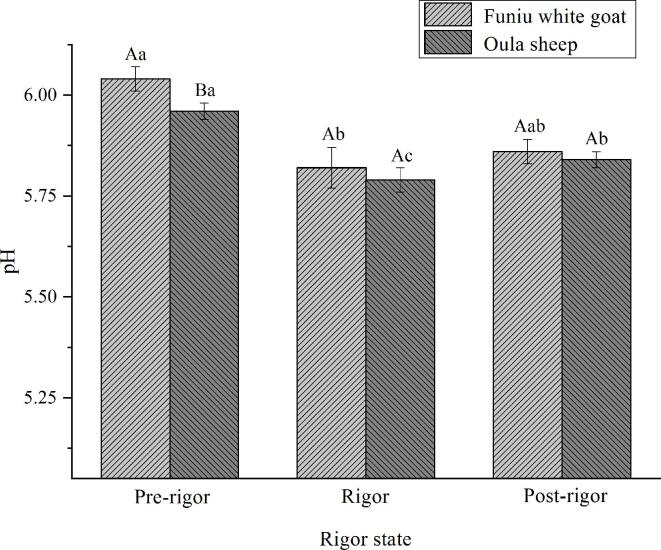
Ph profile of meat batters made from Funiu white goat and Oula sheep meat in different rigor states (n = 9).

pH values of meat batters produced with Funiu white goat and Oula sheep meat are shown in Fig. 1. pH values of Oula sheep meat batter were lower than that of Funiu white goat meat batter. Furthermore, pH values of batters produced with rigor meat were lower than that produced with pre-rigor meat. This could be attributed to the fact that glycolysis is central for the production of adenosine triphosphate (ATP) under the anaerobic environment of skeletal muscle postmortem; however, lactic acid is produced during glycolysis thus reducing meat pH (Lawrie & Ledward, 2006). This finding is in agreement with the results got by v and Geesink et al. (2011), who reported that the pH value of sheep was shown to decrease at the onset of rigor mortis.

### Color

Color is an important quality attributes of meat products, which can significantly affect consumer acceptability (Li et al., 2022). The effect of rigor state on color of Funiu white goat and Oula sheep meat batters is presented in Table 2. L* and *W* values of meat batters made from pre-rigor meat were higher than that of meat batters made from post-rigor meat, which can be attributed to differences in moisture content that influence on the ability of the sample to reflect light. Moreover, b* values of meat batters made from pre-rigor meat were lower than those of meat batters made from post-rigor meat. The results are in agreement with those reported by Ramanathan, Soman, and Faustman (2020), who found that rigor state can influence meat color. Furthermore, Funiu white goat meat batters had higher a* values than Oula sheep meat batters, thereby indicating that Funiu white goat meat could be a suitable raw material for the production of bright-colored meat batters. Similar results were obtained by Ortega, Chito, and Teixeira (2016), who found differences in color characteristics of goat meat and sheep meat, which may be due to the difference in the content of myoglobin and other chromogenic substances in these matrices.

**Table 2:** Color of meat batters made from Funiu white goat and Oula sheep meat in different rigor states (n = 9).

	Funiu white goat			Oula sheep		
	Pre-rigor	Rigor	Post-rigor	Pre-rigor	Rigor	Post-rigor
L*	51.53 ± 0.67^Aab^	52.97 ± 0.38^Aa^	50.30 ± 0.26^Ab^	49.30 ± 0.44^Bb^	51.07 ± 0.49^Aa^	49.43 ± 0.75^Ab^
a*	7.73 ± 0.03^Aa^	6.98 ± 0.17^Ab^	7.14 ± 0.07^Ab^	7.11 ± 0.03^Ba^	6.75 ± 0.04^Bb^	6.56 ± 0.05^Bc^
b*	8.31 ± 0.08^Ab^	8.82 ±0.20^Aa^	8.72 ± 0.15^Aab^	8.12 ± 0.12^Ab^	8.77 ± 0.06^Aa^	8.78 ± 0.01^Aa^
*W*	26.61 ± 0.49^Aa^	26.52 ± 0.64^Aa^	24.15 ± 0.71^Ab^	24.93 ± 0.10^Aa^	24.75 ± 0.33^Ba^	23.11 ± 0.77^Ab^

### WHc

**Fig. 2:**
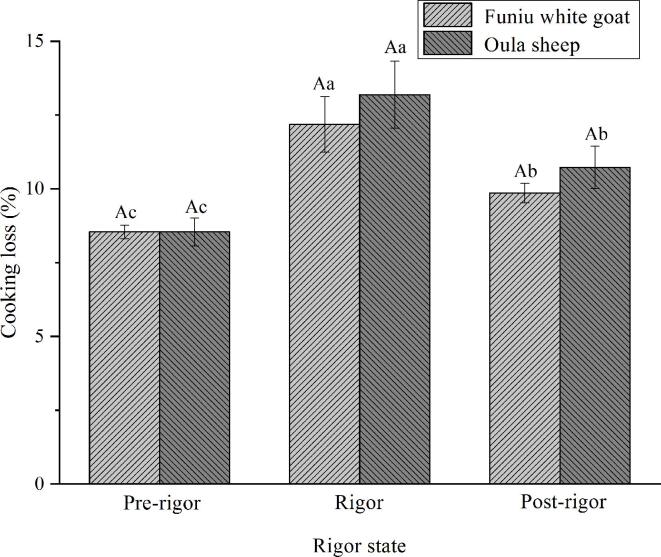

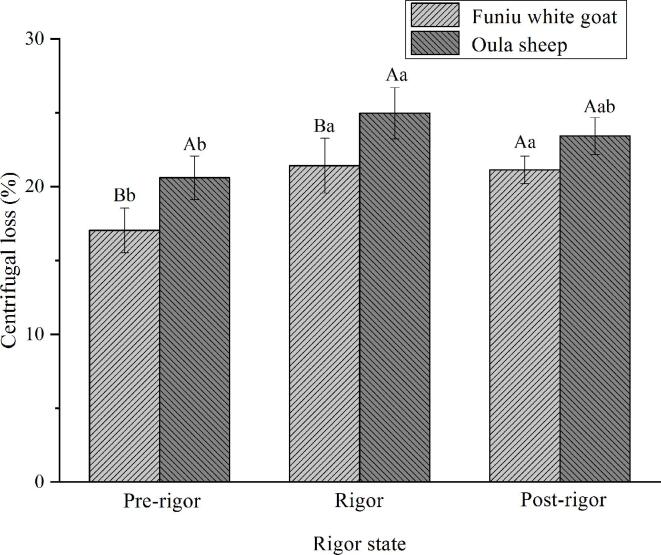
Cooking loss (a) and centrifugal loss (b) of meat batters made from Funiu white goat and Oula sheep meat in different rigor states (n = 9).

Changes in cooking loss (Fig. 2a) and centrifugal loss (Fig. 2b) of meat batters made from Funiu white goat and Oula sheep meat in different rigor states are displayed. These changes means the variance in WHC, which is crucial for the meat quality in processing due to the key role of water played in meat quality (Duun & Rustad, 2007). Increasing in WHC positively influences the meat quality attributes and sale value.

The cooking loss and centrifugal loss of meat batters made from pre-rigor meat were lower than that of meat batters made from post-rigor meat. The results meant the high WHC of meat batters made from pre-rigor meat. It may be because pre-rigor meat had high pH value and a relatively complete organizational structure, which was important for water holding capacity of meat. These results were similar to the reported by Qian et al. (2020), who found that endogenous enzymes released during meat ageing can destroy myofibrillar structure and lead to a bad gel texture. Furthermore, Funiu white goat meat batters had lower cooking loss and centrifugal loss than Oula sheep meat batters. The results meant that Funiu white goat meat is more suitable than Oula sheep meat for meat batters production in terms of WHC.

### LF NMr

**Fig. 3:**
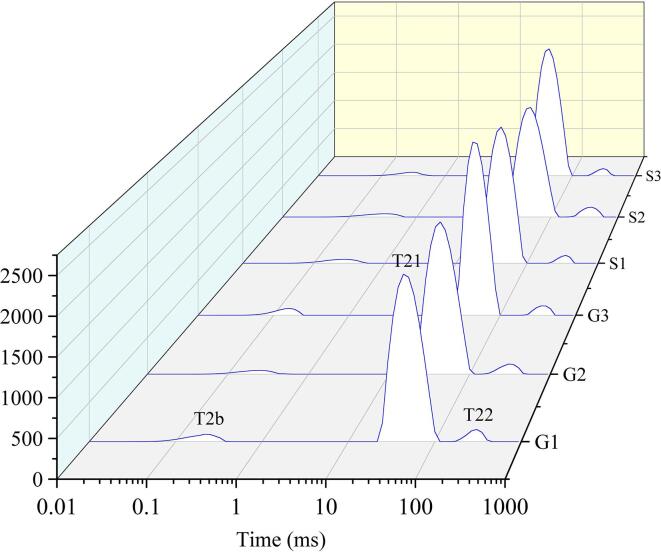

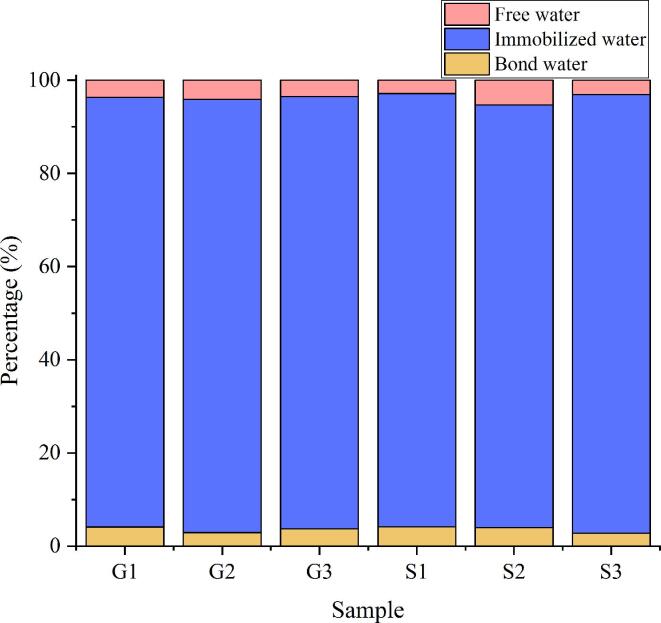
Distribution of LF NMR relaxation times (a) and percentage of three types water (b) of meat batters made from Funiu white goat and Oula sheep meat in different rigor states. (G1, meat batters made from Funiu white goat meat in pre-rigor state; G2, meat batters made from Funiu white goat meat in rigor state; G3, meat batters made from Funiu white goat meat in post-rigor state; S1, meat batters made from Oula sheep meat in pre-rigor state; S2, meat batters made from Oula sheep meat in rigor state; S3, meat batters made from Oula sheep meat in post-rigor state) (n = 9).

LF NMR has been used for evaluating water distribution and mobility. In general, water is present in meat in three distinct forms: free water, immobilized water, and bound water (McDonnell et al., 2013). In this research, three peaks in LF NMR spectra were detected (Fig. 3a), namely, T22, T21, and T2b, which represented free water, immobilized water, and bound water, respectively.

As shown in Fig. 3a, relaxation time of T21 of Oula sheep meat batters was significant shorter than that of Funiu white goat meat batters. Free water content in meat batters obtained from rigor meat was the highest among batters produced with meat in the three rigor states tested (Fig. 3b). These results reflected that WHC of meat batters produced with rigor meat was low, which was mainly due to variation in the capacity of protein molecules bonding water. This could be attributed to the very low pH value of rigor meat. The low pH can reduce the electrostatic repulsion between filaments.,which resulted in the weak binding force between muscle filament and water (Li, He, & Li, 2018). Furthermore, protein degradation could lead to considerable damages to myofibrillar protein and cell membranes, which can further weaken protein-water interaction (Bertram et al., 2002, Puolanne and Halonen, 2010). These could be the reasons underlying high water mobility in meat batters produced with post-rigor meat. In addition, the LF NMR results are in agreement with WHC results in the study.

### Texture properties

Texture is another important parameter for meat batters in terms of quality and acceptability. Texture properties including hardness, springiness, cohesiveness, and chewiness can objectively reflect eating characteristic of meat products (Schreuders, Schlangen, Kyriakopoulou, Boom, & Goot, 2021). Textural properties of meat batters obtained from Funiu white goat and Oula sheep meat are provided in Table 3.

**Table 3:** Texture properties of meat batters made from Funiu white goat and Oula sheep meat in different rigor states (n = 9).

	Funiu white goat			Oula sheep		
	Pre-rigor	Rigor	Post-rigor	Pre-rigor	Rigor	Post-rigor
Hardness (g)	850.55 ± 16.69^Aa^	797.67 ± 11.42^Ab^	788.32 ± 21.93^Ab^	819.73 ± 18.30^Ba^	763.45 ± 11.51^Bb^	769.97 ± 16.91^Ab^
Springiness (%)	53.34 ± 6.10^Aa^	56.74 ± 8.91^Aa^	48.82 ± 2.59^Aa^	48.94 ± 1.84^Aa^	52.00 ± 4.49^Aa^	50.32 ± 3.02^Aa^
Cohesiveness	0.38 ± 0.05^Aa^	0.37 ± 0.04^Aa^	0.34 ± 0.02^Aa^	0.38 ± 0.01^Aa^	0.37 ± 0.03^Aa^	0.39 ± 0.05^Aa^
Chewiness (mJ)	143.05 ± 9.64^Aa^	148.07 ± 9.39^Aa^	124.18 ± 10.10^Ab^	150.76 ± 6.95^Aa^	154.20 ± 11.37^Aa^	116.66 ± 15.34^Ab^

Meat batters produced with pre-rigor meat had higher hardness, springiness, and chewiness compared to batters obtained from post-rigor meat. These results are in agreement with WHC alterations, which indicated that meat batters made from pre-rigor meat had better gelation properties. Similar results were reported by Xue et al. (2018) who described that ageing can significantly influence meat gelation properties. Zheng et al. (2015) also demonstrated that a positive correlation between WHC and texture properties of meat gel network. There was no significantly difference in chewiness between Funiu white goat meat batters and Oula sheep meat batters.

### Microstructure

**Fig. 4:**
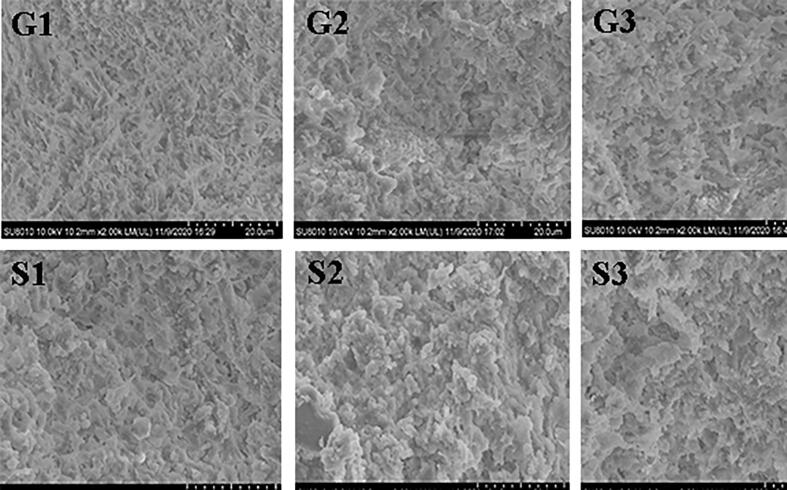
SEM of meat batters made from Funiu white goat and Oula sheep meat in different rigor states. (G1, meat batters made from Funiu white goat meat in pre-rigor state; G2, meat batters made from Funiu white goat meat in rigor state; G3, meat batters made from Funiu white goat meat in post-rigor state; S1, meat batters made from Oula sheep meat in pre-rigor state; S2, meat batters made from Oula sheep meat in rigor state; S3, meat batters made from Oula sheep meat in post-rigor state) (n = 9).

SEM observations of meat batters produced with Funiu white goat and Oula sheep meat are displayed in Fig. 4. Microstructure of meat batters differed considerably; meat batters produced with rigor meat was porous. However, the network of meat batters obtained with pre-rigor meat appeared denser and smoother compared to that of batters produced with rigor meat. The results indicated that rigor mortis had a negative influence on gel formation capacity of meat batter. Despite the presence of breaks, the microstructure of meat batters obtained from post-rigor meat was denser compared to that of batters made from rigor meat, suggesting that ageing can improve to some extent gel formation capacity of meat batters (Hrynets et al., 2014, Zheng et al., 2017). Furthermore, gel networks of Funiu white goat meat batters were smoother than those of Oula sheep meat batters. Collectively, these results are in agreement with WHC and texture properties changes described earlier in the study.

### Rheological

**Fig. 5:**
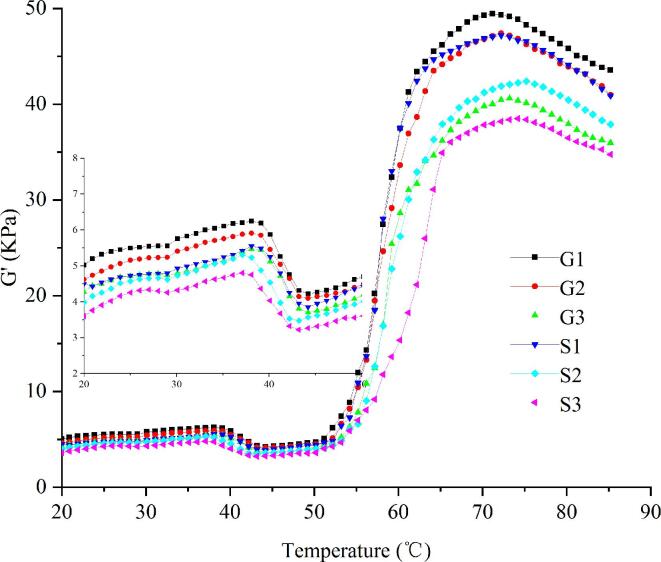
Rheological properties of meat batters made from Funiu white goat and Oula sheep meat in different rigor states. (G1, meat batters made from Funiu white goat meat in pre-rigor state; G2, meat batters made from Funiu white goat meat in rigor state; G3, meat batters made from Funiu white goat meat in post-rigor state; S1, meat batters made from Oula sheep meat in pre-rigor state; S2, meat batters made from Oula sheep meat in rigor state; S3, meat batters made from Oula sheep meat in post-rigor state) (n = 9).

Differences in storage modulus (G′) of meat batters of Funiu white goat and Oula sheep meat are displayed in Fig. 5. G′ indicates energy recovered per cycle of sinusoidal shear deformation, which was related to the elastic gel structure formation (Egelandsdal, Martinsen, & Autio, 1995). In this study, the G′ changes in Funiu white goat and Oula sheep meat batters can be described as four stages: 1) a initial increase at 19 °C to 37 °C; 2) a slight decrease to the minimum at 42 °C or 43 °C; 3) the second increase beginning at 44 °C and reaching to a maximum at 72 °C to 75 °C; 4) the second decrease at final period of heating.

The first G′ peak of Funiu white goat and Oula sheep meat batters appeared at approximately 29 °C, reaching the maximum value at 36.1 °C to 38.0 °C. The peak is formed due to the unfolding and cross-linking of light meromyosin chains (Reed & Park, 2011). Moreover, rigor state did not influence G’ peak temperature but led to a decrease in G’ values. Funiu white goat meat batters had higher G’ values than Oula sheep meat batters. The slight decrease in the G’ value to a minimum of 42 °C or 43 °C could be attributed to the helix-to-coil transformation of meat myosin, which could damage meat batter gel network (Sano, Noguchi, Tsuchiya, & Matsumoto, 1988). Subsequently, G′ values constantly increased and peaked within the range of 72–75 °C due to the cross-linking among meat protein aggregates (Li, He, & Li, 2018). Rigor state could affect the maximum G’ value, and meat batters produced with pre-rigor meat had higher G’ value than that of batters with post-rigor meat. These results are in agreement with findings of textural properties described earlier in this study.

### E-nose

**Fig. 6:**
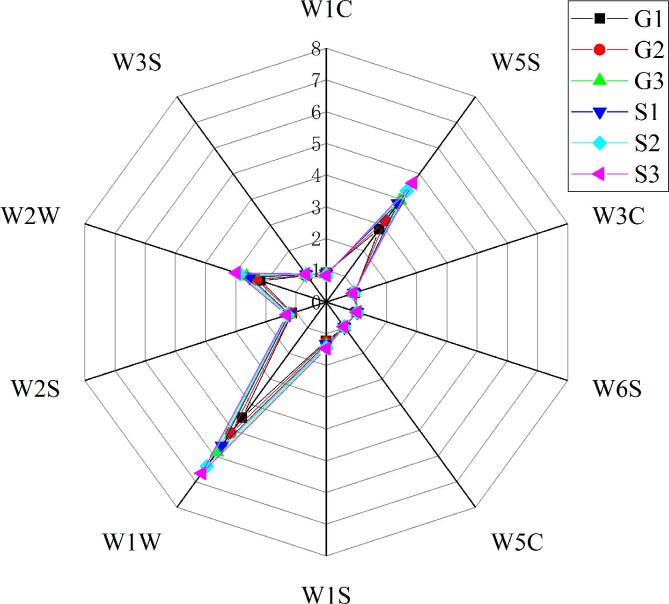
Rad chart of E-nose data of meat batters made from Funiu white goat and Oula sheep meat in different rigor states. (G1, meat batters made from Funiu white goat meat in pre-rigor state; G2, meat batters made from Funiu white goat meat in rigor state; G3, meat batters made from Funiu white goat meat in post-rigor state; S1, meat batters made from Oula sheep meat in pre-rigor state; S2, meat batters made from Oula sheep meat in rigor state; S3, meat batters made from Oula sheep meat in post-rigor state) (n = 9).

The E-nose technology employs a group of sensors that simulate the human nose to distinguish olfactory characteristics of foods, thus providing rapid and overall information about a sample’s volatile substances (Hu, Lu, Guo, & Zhu, 2020). E-nose sensor responses values of meat batters produced with Funiu white goat and Oula sheep meat are presented in Fig. 6.

The response values of sensors W5S, W1W, and W2W of meat batters made from pre-rigor meat were lower than that of meat batters made from post-rigor meat, which thus indicated that volatile compounds were generated during meat ageing because of protein degradation or lipid oxidation (Kitamura et al., 2005). Furthermore, response values of sensors W5C and W1W were lower in Funiu white goat meat batters than Oula sheep meat batters. The detection of organosulfur compounds was significantly different between Funiu white goat and Oula sheep meat batters. Collectively, these results indicated that Funiu white goat and Oula sheep meat batters had distinct odor quality profiles.

### E-tongue

As a kind of detection technology, E-tongue uses multi-sensor array as the basis to identify, analyze, and judge the sample (Banerjee et al., 2016, Zaikuu et al., 2020). Table 4 shows the response values of E-tongue of meat batters of goat and sheep meat in three rigor states.

**Table 4:** Response values of E-tongue of meat batters made from Funiu white goat and Oula sheep meat in different rigor states (n = 9).

	Funiu white goat			Oula sheep		
	Pre-rigor	Rigor	Post-rigor	Pre-rigor	Rigor	Post-rigor
AHS	893.79 ± 11.65^Ab^	942.26 ± 13.05^Aa^	893.33 ± 14.24^Ab^	883.42 ± 14.67^Ab^	954.71 ± 16.65^Aa^	891.65 ± 12.60^Ab^
PKS	811.26 ± 30.46^Ab^	853.36 ± 27.44^Bab^	891.35 ±29.92^Aa^	832.31 ± 27.99^Ab^	905.69 ± 21.72^Aa^	897.23 ± 25.68^Aa^
CTS	1638.69 ± 24.01^Ab^	1776.83 ± 39.63^Aab^	1832.66 ± 50.38^Aa^	1536.48 ± 48.96^Bb^	1792.92 ± 40.20^Aa^	1867.67 ± 51.34^Aa^
NMS	2990.59 ± 22.09^Aa^	3010.12 ± 21.62^Aa^	3002.18 ± 26.60^Aa^	2935.98 ± 27.99^Bb^	3046.58 ± 14.71^Aa^	3031.17 ± 22.35^Aa^
CPS	1272.40 ± 42.72^Ab^	1359.07 ± 31.07^Aa^	1368.22 ± 18.64^Aa^	1301.39 ± 21.89^Ab^	1360.90 ± 28.36^Aa^	1379.05 ± 29.10^Aa^
ANS	3202.63 ± 16.38^Aa^	3229.33 ± 10.64^Aa^	3212.55 ± 20.92^Aa^	3213.01 ± 24.15^Aa^	3177.77 ± 28.64^Bab^	3142.99 ± 22.26^Ab^
SCS	1374.63 ± 21.61^Aa^	1373.10 ± 11.85^Aa^	1375.54 ± 21.40^Aa^	1373.26 ± 11.73^Aa^	1373.41 ± 11.21^Aa^	1372.34 ± 12.52^Aa^

The response values of CPS (universal sensor) of meat batters made from pre-rigor meat were lower than that of meat batters made from post-rigor meat. It may be owing to the protein degradation by μ-calpain and microorganism (Li et al., 2022). Besides, the meat batters made from rigor meat had higher AHS (sourness) value than that made from pre- and post-rigor meat. The result could be explained by the increase in lactate producd by anaerobic glycolysis (Reid et al., 2017). Furthermore, the response values of CTS (saltiness) and NMS (fresh taste) of Funiu white goat meat batters were higher than that of Oula sheep meat batters. The results meant that Funiu white goat and Oula sheep meat batters had distinct taste sense.

## Conclusion

Funiu white goat and Oula sheep meat batters had different gelation and odor properties which were affected by meat rigor state. The content of organosulfur compounds in meat batters differed significantly between batters obtained from pre- and post-rigor meat. Overall, Funiu white goat and Oula sheep can be the good raw materials for meat batters production. Pre-rigor meat from Funiu white goat and Oula sheep were shown to be more suitable for the production of minced meat products. The production of processed products can promote the development of goat and sheep industry in China.

## CRediT authorship contribution statement

**Shaobo Li:** Investigation, Formal analysis, Writing – original draft. **Dequan Zhang:** Project administration, Supervision. **Can Xiang:** Data curation. **Yue Ge:** Data curation. **Huan Liu:** Visualization. **Xiaochun Zheng:** Software. **Li Chen:** Writing – review & editing. **Zhenyu Wang:** Conceptualization, Project administration.

## Declaration of Competing Interest

The authors declare that they have no known competing financial interests or personal relationships that could have appeared to influence the work reported in this paper.

## Data Availability

The data that has been used is confidential.
